# The Inhibitory Effects of *Hericium erinaceus* β-glucan on *in vitro* Starch Digestion

**DOI:** 10.3389/fnut.2020.621131

**Published:** 2021-01-21

**Authors:** Bowen Ma, Tao Feng, Sanfeng Zhang, Haining Zhuang, Da Chen, Lingyun Yao, Jingsong Zhang

**Affiliations:** ^1^National Engineering Research Center of Edible Fungi, Shanghai Academy of Agricultural Sciences, Shanghai, China; ^2^School of Perfume and Aroma Technology, Shanghai Institute of Technology, Shanghai, China; ^3^Department of Food Science and Technology, The Ohio State University, Columbus, OH, United States

**Keywords:** *Hericium erinaceus*, β-glucan, starch digestion, structure, mechanism

## Abstract

β-glucan has attracted extensive attention due to its health promoting effects, such as lowering the blood sugar and lipids levels, and enhancing immunity. In this study, three different β-glucans (HEBG-1, HEBG-2, HEBG-3) were obtained from *Hericium erinaceus* by sodium hydroxide, β-1,3-glucanase and β-1,6-glucanase, respectively. The effects of the glucans on *in vitro* digestion of wheat starch were investigated by Englyst method. We found that addition of HEBGs significantly reduced the digestibility of starch, showing as decreased RDS and pGI, and increased SDS and RS content. In addition, the inhibitory effects positively correlated with the molecular weight of HEBG. The triple helix structure in HEBG plays important roles in inhibiting starch digestion. And β-1,3- glucan showed stronger inhibitor effects than those of β-1,6- glucan. This study unravels the mechanism of HEBG on inhibition of starch digestion and provides a theoretical understanding for the application of edible mushroom β-glucan to the development of low glycemic index starchy foods.

## Introduction

*Hericium erinaceus* is an edible mushroom with health beneficial effects and has attracted extensive research interests (1, 2). The mushroom contains a variety of bioactive components, such as phenolics, steroids, alkaloids, lactones, monounsaturated fatty acids, essential amino acids, polysaccharides, and glycoproteins (3, 4). Mushroom polysaccharides, a type of bioactive carbohydrates isolated from fungal fruiting body, mycelium and fermentation broth of edible fungus (5). They are mainly glucans comprising more than 10 monosaccharides connected by glycosidic bond (6). Of which, (1-3), (1-6)-β-glucan is the main constituent (7). This type of polysaccharide or oligosaccharide have been demonstrated to possess multiple biological functions, such as antitumor and immunomodulation, anti-gastric ulcer, neuroprotection and neuroregeneration, anti-oxidation and hepatoprotection, anti-hyperlipidemia, anti-hyperglycemia, anti-fatigue, and anti-aging (8, 9). A range of methods are available to extract of β-glucan, such as hot water extraction (10), solvent extraction (11), enzymatic extraction (12), and alkali extraction (13). Mushroom β-glucans have also been chemically modified by sulfation and carboxymethylation to improve their functional properties.

Nowadays, with the improvement of living standards and the changes of lifestyle, diabetes poses a serious threat to human health. It is the third most serious chronic non-communicable disease after tumor and cardiovascular diseases (14). Replacing high glycemic index (GI) carbohydrates with low-GI carbohydrates in human diet has been recommended as an efficient way to reduce or prevention the occurrence of diabetes (15). Starch is not only the most important source of carbohydrates for human beings, but also the raw material of many food products (16). Reducing the digestion rate of starch could slow down the increase of postprandial glycemix and plays central role in controlling diabetes and obesity (17). Many compounds have been found to reduce *in vitro* digestions of starch, such as dietary fiber, polyphenols, and fatty acid, etc. The digestion rate of starch in coarse grains and beans containing more dietary fiber was proved to be significantly slow. Englyst method was mainly used to measure starch digestibility indexes (16). According to the time of *in vitro* digestion of starch, starch can be divided into three categories: rapidly digestible starch (RDS), which can be digested quickly in the small intestine, and digestion time <20 min; slowly digestible starch (SDS) refers to those can be fully digested and absorbed in the small intestine but, slowly release energy and help to maintain postprandial blood glucose stability without insulin resistance; resistant starch (RS) refers to that cannot be digested and absorbed in the human small intestine, but can be fermented by microorganisms in the large intestine to maintain the gut health. Some studies have shown that eating foods rich in SDS can effectively prevent chronic diseases such as diabetes. A number of works have confirmed the potential of *H. erinaceus* in the treatment of diabetes (18).

How polysaccharides affect starch digestibility remains inconclusive. To the best of the authors' knowledge, there are no systematic papers in the literature specifically devoted to study the inhibition of edible fungus polysaccharide on starch digestibility. Although much attention has been given to the extraction methods, structural behavior and functional nutrition of *H. erinaceus* β*-gluacn* (2, 19), little research has been done on the mechanism of HEBG and starch digestion is available in literatures. One theory is that polysaccharides either hinder starch digestion by formation of external physical barriers and reduce enzyme binding sites and enzyme catalytic activity (20). Kim et al. studied the relationship between oat β-glucan and starch digestion *in vitro*, and found that GI value was negatively correlated with β-glucan content due to its capacity to alter viscosity of starch, the higher the viscosity, the lower the digestibility of starch (21). In the present study, β-D-glucans were extracted from *H. erinaceus* using alkaline solution or enzymes treatment. Their molecular structure and morphology were analyzed with chemical approaches and atomic force microscope. The effects of the polysaccharides on the *in vitro* digestion of wheat starch were then assessed based on the content of rapidly digestible starch (RDS), slowly digestible starch (SDS) and resistant starch (22), and the predictive glycemic index (pGI).

## Materials and Methods

### Materials

*Hericium erinaceus* was provided by Shanghai Baixin Biotechnology Co., China. endo-β-(1-3)-D-glucanase (EC 3.2.1.39, 2 units/mg) from *Helix pomatia*, endo-β-(1-6)-D-glucanase (EC 3.2.1.75, 35 units/mg) from *Talaromyces cellulolyticus*, pepsin (EC 3.4.23.1, 51 units/mg) from porcine stomach mucosa, invertase Grade VII (EC 3.2.1.26, 300 units/mg) from baker's yeast (*S. cerevisiae*), thermostable α-amylase (EC 3.2.1.1, 21 units/mg) from *Bacillus licheniformis*, they were purchased from Sigma-Aldrich Chemical Co. (St. Louis, MO, USA). Wheat starch (~98.5% starch content) was provided by Henan Kangdi Food Tech Co., Ltd, China. All the water used are deionized.

#### Preparation of HEBG

Extraction of HEBG was conducted according to previous methods (23) with slight modifications. One Kilogram *Hericium erinaceus* was weighted into 15 L water, mixed and heated to 100°C for 2 h, under stirring followed by cooling at room temperature. The solution was filtered through 4 layers of cloth (200 mesh). The retentate was heated again as before, concentrated to ~500 mL (dry weight of fruiting body: volume of crude extract), and centrifuged (25°C, 12,840 × g, 15 min). The precipitate was collected and washed with 20% ethanol, centrifuged and repeated 3 more times to obtain white jelly-like precipitate. The solution was then dialyzed against water for 3 days (3500 Da cut-off membrane), during which the water was changed 6 times and the retentate was freeze-dried to obtain HEBG.

#### Preparation of HEBG-1 With Sodium Hydroxide

Briefly, 1 g HEBG was weighted into the 20 mL water and magnetically stirred at 60°C for 5 h. 1M sodium hydroxide solution was added, mixed, sealed and shaken intermittently for 24 h at room temperature. The solution was dialyzed and freeze dried as above, and named as HEBG-1.

#### Preparation of HEBG-2 and HEBG-3 With Enzymes

HEBG (100 mg) were mixed with water (50 μL) and magnetically stirred at 70°C to completely dissolve the polysaccharides. The glucan solution was cooled down to 50°C followed by addition of β-(1-3)-D glucanase or β-(1-6)-D-glucanase (3 mg). After 50 min hydrolysis, the enzymes were denatured by heating in a boiled water bath for 10 min. The hydrolysates were cooled down at room temperature and freeze-dried. The HEBG hydrolysates from β-(1-3)-D glucanase and β-(1-6)-D-glucanase was named as HEBG-2 and HEBG-3, respectively.

### Characterization of HEBGs

#### Determination of Molecular Weight

HEBG-1, 2, or 3 (2 mg) was dissolved in pH 7 salt solution (1 mL) containing 0.05 mol/L NaH_2_PO_4_ and 0.15 mol/LNaNO_3_. They were filtered through 0.22 μm filter prior to loaded into high performance size exclusion chromatography (HPSEC) system equipped with PWXL3000 and TSKPWCL4000 series gel column. The injection volume was 20 μL. The flow rate was 0.5 mL/min, the column temperature was maintained at 35°C and the laser detector light source wavelength was 623.8 nm (24).

#### Determination of Tertiary Structure of HEBG by Using Congo Red

The mixtures of HEBG (5 mg) and distilled water (2 mL) were stirred at 60°C for 3 h until completely dissolved. Congo red solution (2.0 mL, 80 μmol/L) was then added and mixed thoroughly (25). 1M NaOH was then added to mixture till the final concentration of NaOH in the solution reaches 0.1, 0.2, 0.3, 0.4, and 0.5 mol/L, respectively. At each NaOH concentration, the solutions were scanned by UV-vis spectrophotometer and the maximum absorption wavelength was recorded.

#### Periodate Oxidation, Smith Degradation, and Methylation Analysis

The standard curve drawing method of sodium periodate consumption (26): Sodium periodate solution (0.15 mol/L) and sodium iodate solution (0.15 mol/L) were mixed evenly in different proportions. The solution of 0.2 mL was diluted to 50 mL and monitored at 223 nm to determine the absorbance. The concentration of sodium periodate in the mixed solution was taken as abscissa (x) and absorbance as ordinate (y). After testing, the standard curve fitting equation was y = 44.107x + 0.2837, *R*^2^ = 0.9977.

Periodate oxidation: HEBG-1, 2, or 3 (50 mg) and sodium periodate solution (50 mL, 0.015 mol/L) were mixed at 4°C. 0.1 mL of samples were taken from the mixture every 24 h and diluted to 25 mL with water. The absorbance of the diluent was monitored at 223 nm until a maximum value was reached. The sodium periodate consumption of HEBG was calculated by the standard curve. Then 8 mL periodate oxidation solution and two drops of ethylene glycol was added to the original mixture to terminate the reaction. The yield of formed formic acid was titrated with 40% NaOH after adding two drops of phenolphthalein (27).

Smith degradation was conducted according to Kocharova (28). The HEBG-1, 2, and 3 solution was firstly oxidized by periodate. Ethylene glycol was then added to remove excess periodate followed by dialysis against water for 48 h. Subsequently, the solution was adjusted to pH = 6~7 with 50% acetic acid and mixed NaBH_4_ holding 24 h. Then the solution dialyzed continuously for 2 days. Polysaccharide alcohol product was obtained from above solution by freeze-drying, which were analyzed by aldononitrile acetate precolumn-derivatization gas chromatography (29).

Methylation analysis of the glucans were conducted according to method of Panda et al. (30). HEBG-1, 2, or 3 (10 mg) was completely dissolved in anhydrous dimethyl sulfoxide (1 mL) in a tube with headspace filled with N_2_. Sodium hydroxide pellet (30 mg) was then quickly added and stirred for 3 h. Methyl iodide (1 mL) was slowly added to the tube on ice bath with a flow of N_2_. Be operated away from light, stirred by magnetic force at room temperature for 1 h and then removed moisture by anhydrous Na_2_SO_4_ column. The methylated glucans were hydrolyzed with trifluoroacetic acid (4 mol/L), reduced with sodium boron deuteride for 3 h, and acetylated with 0.5 mL acetic anhydride at 100°C for 1 h. The formed alditol acetate was dissolved in extracted with dichloromethane and analyzed by GC-MS (Japan shimadzu co., Japan) equipped with a HP-5MS quartz capillary column (30 m × 250 μm × 0.25 μm). The condition was set as: column initial temperature 160°C, 2°C/min ramping rate to 240°C; injection port temperature 250°C, carrier gas was N_2_, with a flow rate 1 mL/min. Sample injection volume was 2 μL.

### *In vitro* Digestion Experiment

The content of enzymatically hydrolyzed glucose was determined based on Zhuang et al. (29) with minor modification. The mixtures of wheat starch (100 mg) and HEBG-1, 2, or 3 (20 mg) were dispersed in 50 mL beaker with distilled water (2 mL), heated in a boiling water bath under stir for 30 min and cooled down at 37°C water bath. Pepsin solution (4 mL, 5 mg/mL) was added to the mixture and stirred at 37°C for 30 min. Two milliliter acetic acid buffer (0.5 mol/L, pH = 5) and six glass beads were added, vortexed, and placed on 37°C water bath for 30 min under shaken (200 r/min). Afterwards, complex enzyme solution (thermostable α-amylase and starch transglucosidase) was added, 50 μL of hydrolysates was sampled at 0, 20, 30, 60, 90, 120, 180, 240 min, respectively. The values of RDS, SDS, and RS was calculated according to the following equations (16).

$$\begin{array}{l} C\left(\%\right)=\left(G_{t}-G_{0}\right)\times 0.9/TS\times 100\\ RDS\left(\%\right)=\left(G_{20}-G_{0}\right)\times 0.9/TS\times 100\\ SDS\left(\%\right)=\left(G_{120}-G_{20}\right)\times 0.9\times 100\\ RS\left(\%\right)=\left(G_{20}-G_{0}\right)\times 0.9/TS\times 100 \end{array}$$

C is the digestibility of wheat starch, G_t_ is the glucose content of wheat starch released at t min of hydrolysis, G_0_ is the free glucose content before digestion, TS (%) is the proportion of total dry weight (Mg) of wheat starch to total weight in each sample.

The predicted glycemic index (pGI) was calculated using the equation below (22):

$$\begin{array}{l} pGI=39.21+0.803\times \left(H_{90}\right) \end{array}$$

which (H_90_) is the percentage of total starch hydrolyzed for 90 min.

### Atomic Force Microscope Observation

HEBG-1, 2, and 3 (1 mg) was dissolved in 5 mL water, centrifuged, and the supernatant was diluted to a concentration of 1 μ g/mL HEBG (31). The diluent (3.0 μL) was deposited on the freshly cleaved mica sheet and dried at room temperature. The samples were imaged with Nano Scope IIIa atomic force microscope (Digital instruments co, USA) under tapping mode with a resonance frequency of 2.0 kHz. AFM images were analyzed and processed by using Nano Scope Analysis software (32).

### Statistical Analysis

All measurements were done in triplicates and results were presented as mean ± standard deviation (SD) (*n* = 3). Values with different letters in the same column differ significantly (*P* < 0.05) according to Duncan's multiple range test. The datas were analyzed with Origin (Version 9.0, Origin Lab Co., USA) and SPSS was used to conduct the significant analysis between samples (Version. 17.0 software, Chicago, IL, USA).

## Results and Discussion

### Characterization of HEBG-1, HEBG-2, and HEBG-3

**Figure 1:**
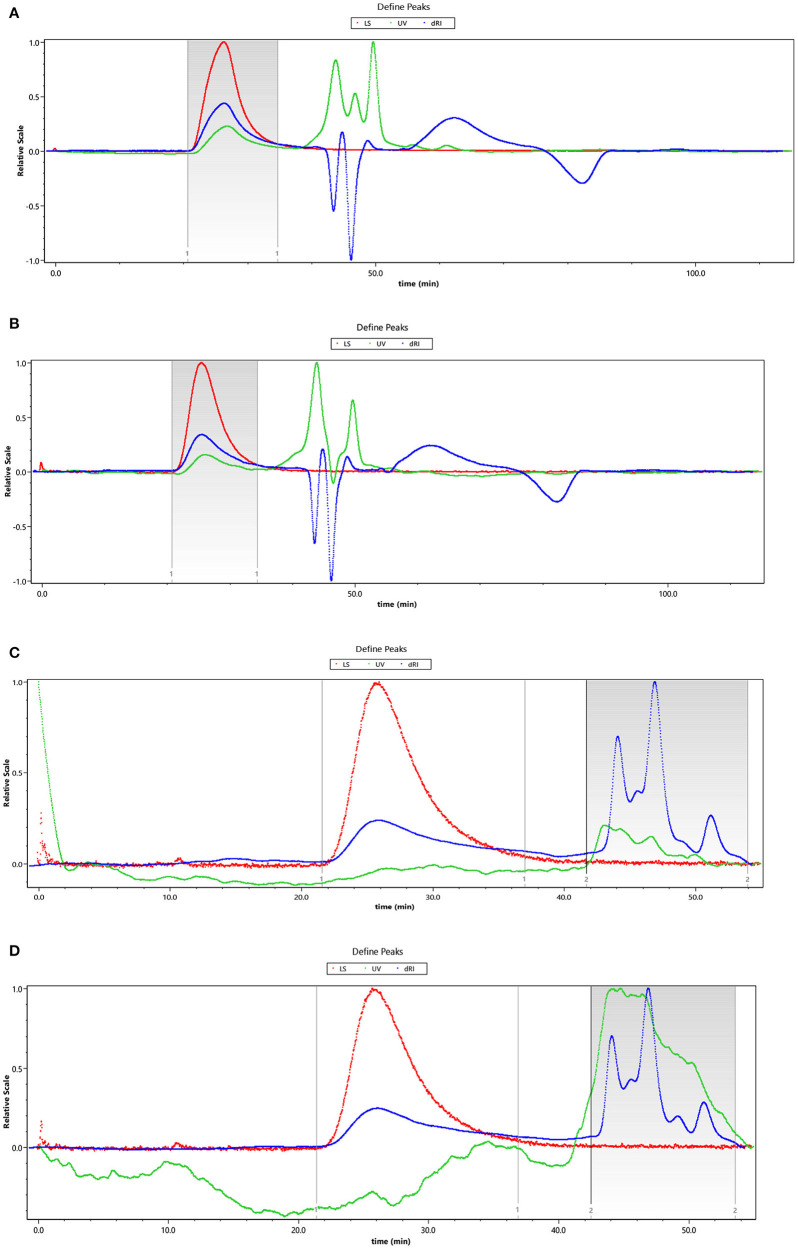
The HPSEC chromatogram of HEBG **(A)**, HEBG-1 **(B)**, HEBG-2 **(C)**, and HEBG-3 **(D)**.

The HEBG is a mixture of glucans with different degrees of polymerization. The molecular weights of HEBG, HEBG-1, 2 and 3 were determined by HPSEC-MALLS-RI. The HPSEC chromatogram of HEBGs (Figures 1A–D) showed a single symmetrical peak, suggesting the HEBGs has narrow range of degrees of polysaccharides. Table 1 shows the molecular weight of HEBG-1 is similar to that of HEBG, suggesting HEBG is relatively stable against 1M sodium hydroxide solution. The Mw of HEBG-2 and HEBG-3 in buffer were estimated to be 4.105 × 10^5^ and 4.573 × 10^5^ g/mol, and were much smaller than HEBG and HEBG-1. This is mainly attributed to degradation of the glucans by glucanases. Similar molecular weight of HEBG-2 and HEBG-3 implies the proportion of 1,3-glycosidic linkage is similar to that of 1,6-glycosidic in HEBG. Further analysis of the polydisperse index (Ratio of Mw to Mn), which can be used to assess the dispersion of the polymersization of the sample. The value close to 1 indicate high homogenous. The value increases from 1.07 in HEBG-1 to 1.54 to HEBG-3, indicating the degree of polymerizations distribute more widely using enzyme treatment of HEBG than using alkaline treatment.

**Table 1:** Molecular weight and polydisperse index of HEBG, HEBG-1, 2, and 3.

**Sample**	**Molecular weights (Mw) (g/mol)**	**Number-average molecular weight (Mw) (g/mol)**	**Polydisperse index (Mw/Mn)**
HEBG	7.504 × 10^5^	6.927 × 10^5^	1.083
HEBG-1	7.300 × 10^5^	6.797 × 10^5^	1.074
HEBG-2	4.105 × 10^5^	3.479 × 10^5^	1.179
HEBG-3	4.573 × 10^5^	2.969 × 10^5^	1.540

### Periodate Oxidation and Smith Degradation of HEBGs

To further elucidate its structure, degradation and methylation experiments were conducted. After mixing sodium periodate and sodium iodate in different proportions, the absorbance was measured at 223 nm, and the standard curve of periodate consumption was obtained (26). The calculation results of periodate consumption were shown in Table 2.

**Table 2:** Experimental data of periodate oxidation of HEBG-1, 2, and 3.

**Sample**	**Weight/mg**	**Time/d**	**Periodate consumption/mmol**	**Formic acid production/mmol**
HEBG	25	10	400.6	42.3
HEBG-1	25	10	437.0	37.1
HEBG-2	25	10	111.5	22.4
HEBG-3	25	10	119.9	11.1

It can be found from the Table 2 that both polysaccharides can produce formic acid, and the amount of formic acid produced by HEBG-2 is more than that of HEBG-3. This suggests that the HEBG-2 contain more 1→6 glycosidic linkage, and the degree of enzymatic hydrolysis is comparatively higher. The HEBG-2 was treated with β-1, 3-glucanase, and HEBG-3 with beta16glucanase, it is obvious that the relative amount of 16 linkages is higher in hegb2 when compared to hebg3, the results from periodate oxidation are in accordance with this fact and in accordance to the methylation analysis results present in Table 3.

**Table 3:** Glycosylic linkage analysis of HEBG-1, 2, and 3.

**Sample**	**Time/min**	**Linkage**	**Methylated sugar**	**Molar percentage %**
HEBG-1	9.779	1-Glc	2,3,4,6-Me4-Glc	8.5^a^
HEBG-1	11.547	1,3-Glc	2,4,6-Me3-Glc	3.6^a^
HEBG-1	13.833	1,6-Glc	2,3,4-Me3-Glc	12.2^a^
HEBG-1	17.625	1,3,6-Glc	2,4-Me2-Glc	63.4^a^
HEBG-2	16.376	1-Glc	2,3,4,6-Me4-Glc	61.5^b^
HEBG-2	23.692	1,6-Glc	2,3,4-Me3-Glc	23.9^b^
HEBG-2	30.373	1,3,6-Glc	2,4-Me2-Glc	14.6^b^
HEBG-3	15.539	1-Glc	2,3,4,6-Me4-Glc	17.9^c^
HEBG-3	20.281	1,3-Glc	2,4,6-Me3-Glc	64.9^c^
HEBG-3	29.865	1,3,6-Glc	2,4-Me2-Glc	17.2^c^

*The molar percentage is the proportion of the normalized residues to the moles of all residues*.

a *denotes normalization in HEBG-1 with the proportion of 1,6-Glc*.

b *denotes normalization in HEBG-2 with the proportion of 1, 3, 6-Glc*.

c *denotes normalization in HEBG-3 with the proportion of 1, 3, 6-Glc*.

**Figure 2:**
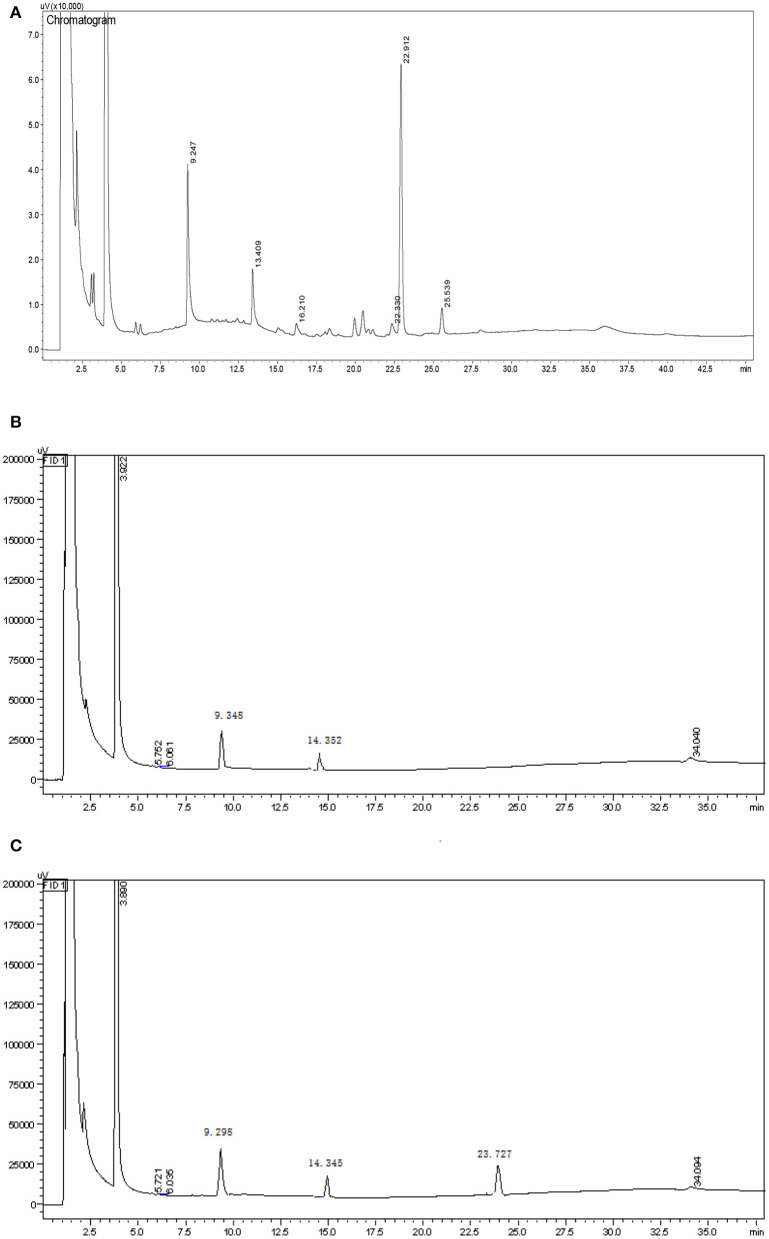
GC chromatogram of the Smith reaction products of HEBG-1 **(A)**, HEBG-2 **(B)**, and HEBG-3 **(C)**.

Smith degradation of the HEBG-1 (-2,-3) was shown in Figures 2A–C. The three peaks at 9.247, 13.409, and 22.912 min are glycerol, erythritol and glucose in Figure 2A, respectively. The existence of glycerol and erythritol indicated the presence of 1, 2-, 1, 4-, or 1, 6- glycosidic linkages in the structure. While formic acid is formed in periodate oxidation test, indicated that the polysaccharides contain 1, 6- glycosidic linkage. A large amount of glucose was also detected in the product, indicating that there were mainly glycosyl groups bonded at 1→3 position. Therefore, it can be inferred that the main chain of HEBG is composed of 1, 3-Glc and the branched chain is composed of 1, 6-Glc.

The product of HEBG-2 has only two peaks, 9.348 min (glycerol) and 14.352 min (erythritol), with a weight ratio of 3.18: 1. For HEBG-3, it has three peaks of 9.298 min (glycerol), 14.345 min (erythritol) and 23.727 min (glucose). No peak of glucose in Figure 2A indicates that the enzymatic hydrolysis reaction is relatively complete. The appearance of glycerol and erythritol chromatographic peaks suggests 1, 2- or 1, 4- and 1, 6- may be the glycosidic linkages in HEBG-2. However, formic acid was formed in periodate oxidation experiment, which indicated that there were 1, 6- glycosidic bonds in the polysaccharides. On this basis, 1, 6- Glc are the main glycosidic linkages in HEBG-2. By contrast, the glucose peak appeared in the HEBG-3 (Figure 2B) concludes 1→3 glycosidic linkages are the dominant linkages.

### Results of Methylation Reaction of HEBGs

Because glucanases hydrolyzes specific glycosidic linkages in polysaccharides, there is no characteristic peak in the degraded gas chromatography. The GC-MS statistics of HEBGs are summarized in Table 3. The methylation products of HEBG-2 are mainly composed of 1-Glc, 1, 6-Glc and 1, 3, 6-Glc, and the proportion between them is 4.20: 1.63: 1. No 1, 3-Glc glycosidic is present in the chromatogram and agrees with the results of Periodic acid and Smith degradation experiments. The methylation products of HEBG-3 are mainly 1-Glc, 1, 3-Glc, and 1, 3, 6-Glc, with a molar ratio of 1.04: 3.77: 1. No 1, 6-Glc glycosidic linkage was detected. Based on the above results, it can be seen that the main component of HEBG-2 is β-1, 6-glucan, and HEBG-3 is β-1, 3-glucan.

### Results of Congo Red of HEBGs

**Figure 3:**
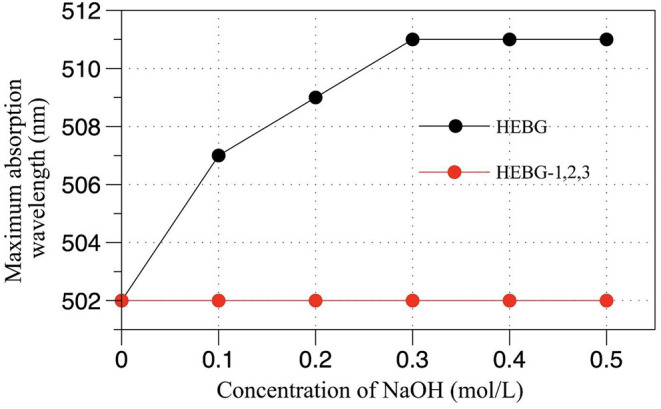
Effect of NaOH concentration on maximum absorption wavelength of Congo red-BCP complex.

The triple helix structure of polysaccharides an important for its biological activity (33). As can be seen from Figure 3, compared with the HEBG whose maximum absorption wavelength increases with NaOH concentration, those of HEBG-1 remains the same at different concentration of NaOH. For HEBG-2 and HEBG-3, they have the same results as HEBG-1. This indicates no triple helix structure exists in HEBG-1, 2, and 3. As HEBG and HEBG-1 has similar molecular weight, so sodium hydroxide only destroys the triple helix structure and has no effect on breaking down the glycosylic bonds.

### Microstructure of HEBG, HEBG-1, 2, and 3

The spatial configuration of polysaccharides correlates with their biological activity. Atomic force microscope (AFM) enables to examine the structure of polysaccharides at nanoscale, which helps to build the connection with its chemical properties (34).

**Figure 4:**
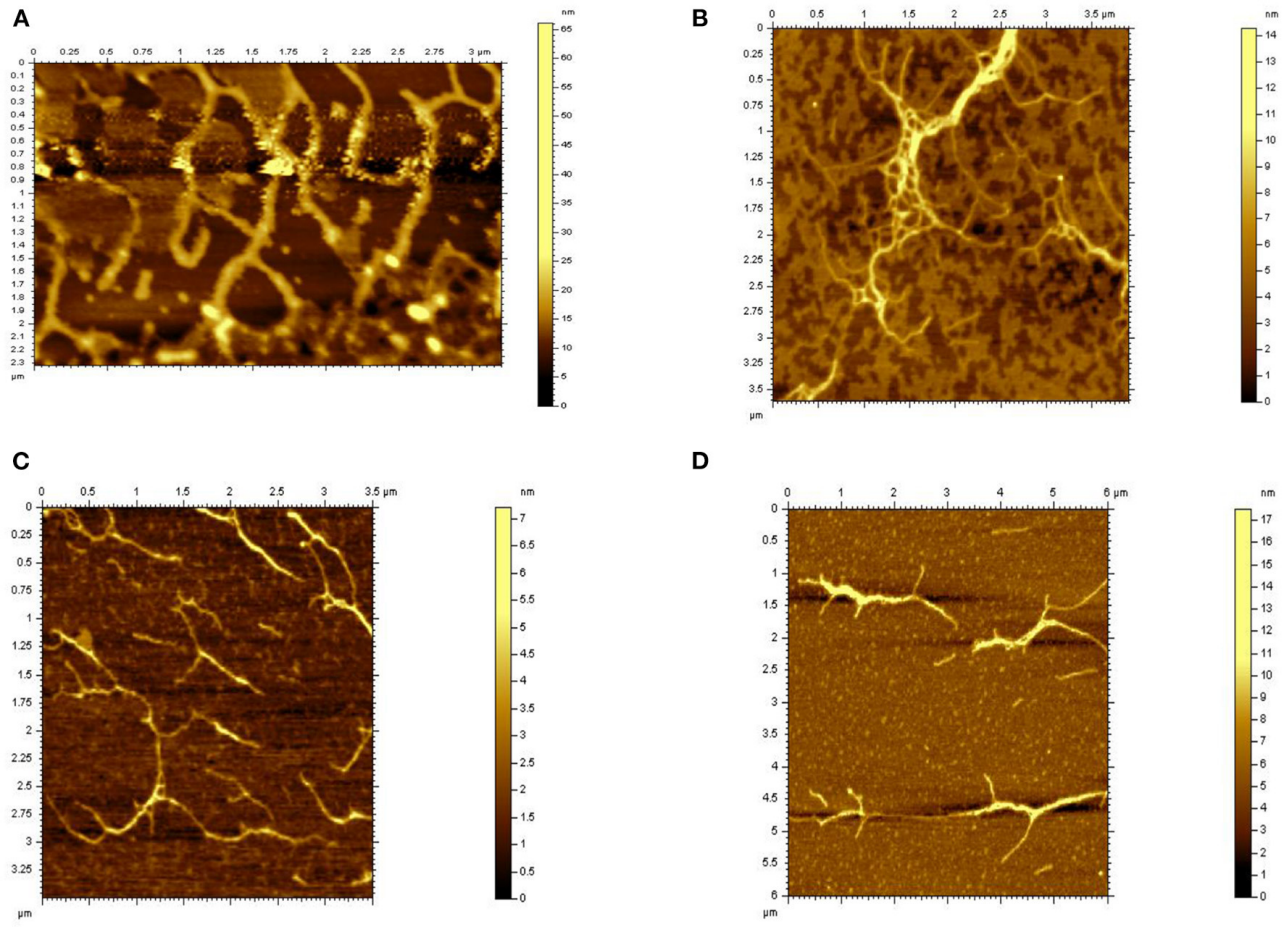
AFM microscopy of HEBGs [**(A)** is HEBG; **(B)** is HEBG-1; **(C)** is HEBG-2; **(D)** is HEBG-3].

The typical microstructure of glucans of four kinds of *Hericium erinaceus* glucans indicating the structure of molecule and space upon different treatments from Figure 4. Untreated glucan (HEBG) (Figure 4A) shows cluster of aggregates, which were scattered heterogeneously throughout the system. The molecular chain diameter of HEBG-1and HEBG are ~0.2 μm, with a height of 6~8 nm. Whereas, HEBG-1 shows a state of aggregation accompanied by stretching (Figure 4B) compared to those of HEBG. The alkali solution can readily cause the cell wall to swell by disrupting the hydrogen bonds (35). This may occur in the HEBG-1. Similar structures were seen for HEBG-2, 3 (Figures 4C,D). Their molecular chain is thinner and shorter, with less branch than those of HEBG and HEBG-1. This indicates some branches were removed during enzymatic hydrolysis, and the polysaccharide conformation in solution has also changed.

It is speculated that the chains of polysaccharides are interacted via mainly hydrogen bonds. When the glycosidic linkage is cleaved by enzymatic hydrolysis, the density of hydrogen bonds between chains becomes smaller and the domain structure becomes larger. The phenomenon was consistent with the Agbenorhevis' study (36). The smaller the molecular weight, the lower the degree of polymerization. Atomic force microscope imaging further explains the changes of the microstructure of polysaccharide aggregates after enzymatic hydrolysis of polysaccharide glycosidic bonds.

### *In vitro* Starch Digestibility

**Figure 5:**
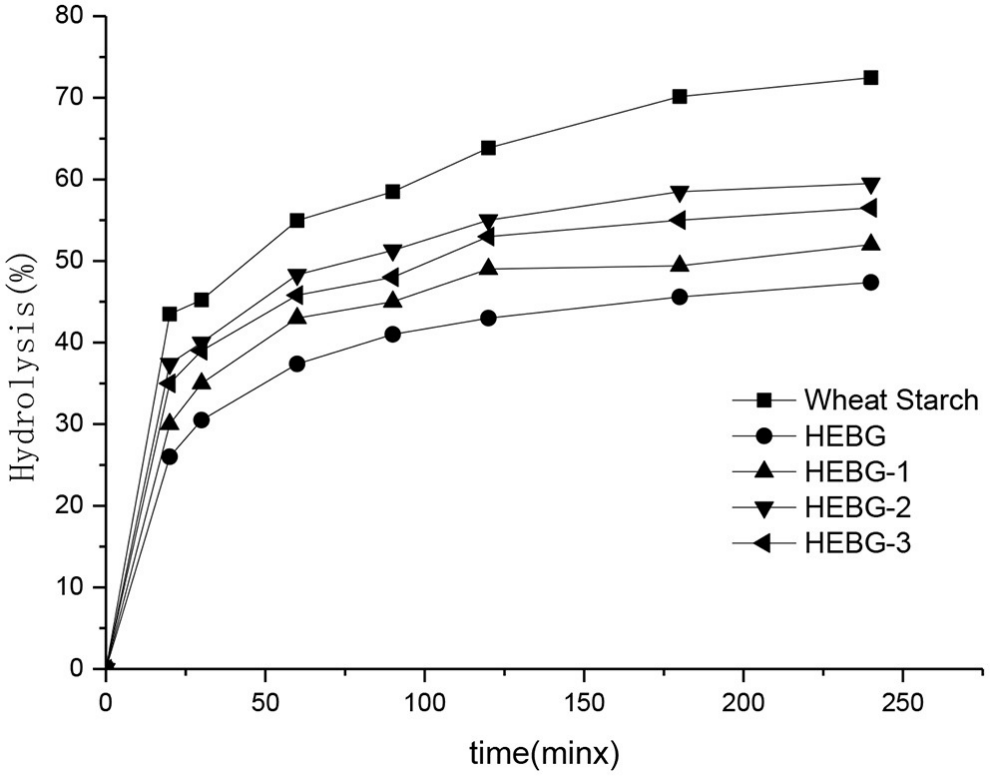
Effects of different Hericium erinaceus β-glucans on *in vitro* digestion of wheat starch.

The effect of different HEBGs on the digestion of wheat starch *in vitro* was studied by measuring the enzymatic hydrolysis rate of wheat starch. As shown in Figure 5, the degree of enzymatic hydrolysis of all samples increases gradually with time, and the digestibility of wheat starch without glucan is the highest. The wheat starch digestibility is more than 70% after 180 min hydrolysis, however, <60% was observed when HEGBs was added. HEBG has the best inhibitory effect on starch digestion, followed by HEBG-1, HEBG-3, and finally HEBG-2. One possible reason for this is the cavity of the triple helix structure of HEBG helps to trap the hydrolyzed starch fragments, thus inhibiting starch digestion (37). The molecular weights of HEBG and HEBG-1 are higher than those of HEBG-2 and HEBG-3. The higher molecular weight and complex structure might be an inducement of starch being surrounded, thus inhibiting the digestion of starch.

The main composition of HEBG-3 is β-1, 3-glucan, so it can be inferred that in the main chain structure of β-glucan of *Hericium erinaceus* β-1, 3-glucan has a greater inhibitory effect on starch digestion in the main chain structure of β-glucan of *Hericium erinaceus*, or it may be that the molecular length of glucan is longer than that of HEBG-2 molecular chain and has a stronger cross-linking effect with starch. This is similar to the result that oat β-glucan without branching structure can also inhibit starch digestion, which was studied by Zhang (38).

### Effects of HEBGs on Starch Nutrition Fragments and pGI Value of Wheat

The TS, RDS, SDS, RS, and pGI values of five groups of samples are shown in Table 4. The addition of HEBG to wheat starch gradually increases the content of SDS and RS, while decreases the values of RDS and pGI. The order of molecular weight of the four groups was HEBG > HEBG-1 > HEBG-3 > HEBG-2. The corresponding RDS (19.34) and pGI (62.56) of HEBG with the highest molecular weight were significantly lower than those of wheat starch without polysaccharides. It is also directly proved that HEBG can significantly inhibit *in vitro* starch digestion. This result is consistent with the interaction between several different β-glucans and other food ingredients that may inhibit starch digestibility and reduce blood sugar response (39, 40). All of them could potentially reduce the peak blood glucose response of food.

**Table 4:** TS, RDS, SDS, RS, and pGI values of wheat starch and wheat starch-HEBGs mixture.

**Sample**	**TS (%)**	**RDS (%)**	**SDS (%)**	**RS (%)**	**pGI**
1 Wheat Starch	98.50 ± 0.53^a^	43.49 ± 0.40^a^	18.35 ± 0.52^a^	38.16 ± 0.34^a^	86.17 ± 0.51^a^
2 HEBG+Wheat Starch	79.10 ± 0.38^b^	19.34 ± 0.35^b^	28.53 ± 0.35^b^	52.13 ± 0.25^b^	62.56 ± 0.45^b^
3 HEBG-1+Wheat Starch	78.87 ± 0.51^b^	22.25 ± 0.37^c^	26.47 ± 0.54^c^	51.28 ± 0.56^c^	65.33 ± 0.49^c^
4 HEBG-2+Wheat Starch	78.93 ± 0.47^b^	27.40 ± 0.53^d^	23.28 ± 0.37^d^	49.32 ± 0.47^d^	70.32 ± 0.25^d^
5 HEBG-3+Wheat Starch	79.05 ± 0.62^b^	25.08 ± 41^e^	24.63 ± 0.49^d^	50.29 ± 0.62^d^	68.79 ± 0.43^e^

*Values with different letters in the same column differ significantly (P < 0.05)*.

## Conclusion

In this study, three different types of HEBGs (HEBG-1, HEBG-2, HEBG-3) were prepared from *Hericium erinaceus* by sodium hydroxide and enzymatic hydrolysis. Their Mw is 7.300 × 10^5^, 4.105 × 10^5^, 4.573 × 10^5^, respectively, and the HEBG is 7.504 × 10^5^. By contrast, HEBG treated with sodium hydroxide will not degrade polysaccharides or change the molecular weight, but enzymatic treatment reduces the molecular weight, and the difference of enzyme leads to different molecular weight changes. It is speculated that the reason is that enzymatic hydrolysis destroys different linkage between molecules. It is inferred that the main component of HEBG-2 is β-1, 6-glucan and the main component of HEBG-3 is β-1, 3-glucan. Through atomic force microscope (AFM), it was directly observed that HEBG was curled and folded, accompanied by filamentous and random coil structure, but the branched chain and conformation of HEBG-2 or 3, with obvious branching characteristics and regular multi-branch juxtaposition structure. The triple helix structure in HEBG plays an important role in inhibiting starch digestion. We found the addition of *Hericium erinaceus* β-glucan could significantly reduce the digestibility of starch, which decreased the values of RDS and PGI, and increased the content of SDS and RS. The glucans treated by enzymatic hydrolysis also showed differences in the starch digestibility, indicating that digestibility inhibition of β-1, 3 linkage on starch digestion was stronger than that of β-1, 6 linkage. Foods added with HEBG can effectively reduce the level of GI, intake of blood sugar and prevent the occurrence of diabetes. To explore the interaction mechanism between β-glucan and starch and reveal its mechanism of inhibiting starch digestion, which provides a basis for the application of β-glucan in function food and health products. This study unravels the mechanism of HEBG on inhibition of starch digestion and provides a theoretical understanding for the application of edible mushroom β-glucan to the development of low glycemic index starchy foods.

## Data Availability Statement

The original contributions presented in the study are included in the article/supplementary materials, further inquiries can be directed to the corresponding author/s.

## Author Contributions

LY and JZ: conceptualization. SZ: methodology. DC: formal analysis. BM: data curation and writing—original draft preparation. TF: writing—review and editing. HZ and JZ: funding acquisition. All authors contributed to the article and approved the submitted version.

## Conflict of Interest

The authors declare that the research was conducted in the absence of any commercial or financial relationships that could be construed as a potential conflict of interest.

## References

[1] Khan MA Tania M Liu R Rahman MM *Hericium erinaceus*: an edible mushroom with medicinal values. J Complement Integr Med. (2013) 10:253–8. 10.1515/jcim-2013-0001 23735479

[2] He X Wang X Fang J Chang Y Ning N Guo H. Structures, biological activities, and industrial applications of the polysaccharides from *Hericium erinaceus* (Lion's Mane) mushroom: a review. Int J Biol Macromol. (2017) 97:228–37. 10.1016/j.ijbiomac.2017.01.040 28087447

[3] Friedman M ChemInform abstract: chemistry, nutrition, and health-promoting properties of *Hericium Erinaceus* (lion′s mane) mushroom fruiting bodies and mycelia and their bioactive compounds. Cheminform. (2015) 46 10.1002/chin.201541264 26244378

[4] Zhang C Cao C Kubo M Harada K Yan X Fukuyama Y. Chemical constituents from *Hericium erinaceus* promote neuronal survival and potentiate neurite outgrowth via the TrkA/Erk1/2 pathway. Int J Mol Sci. (2017) 18:1659. 10.3390/ijms18081659 28758954 PMC5578049

[5] Yin C Noratto G Fan X Chen Z Yao F Shi D. The impact of mushroom polysaccharides on gut microbiota and its beneficial effects to host: a review. Carbohydr Polym. (2020) 250:116942. 10.1016/j.carbpol.2020.116942 33049854

[6] Zhao S Gao Q Rong C Wang S Zhao Z Liu Y. Immunomodulatory effects of edible and medicinal mushrooms and their bioactive immunoregulatory products. J Fungi. (2020) 6:269. 10.3390/jof6040269 33171663 PMC7712035

[7] Kim J Lee S Bae I Park H Gyu Lee H Lee S. (1-3)(1-6)-β-glucan-enriched materials from *Lentinus edodes* mushroom as a high-fibre and low-calorie flour substitute for baked foods. J Sic Food Agric. (2011) 91:1915–9. 10.1002/jsfa.4409 21480277

[8] Liang B Guo Z Xie F Zhao A. Antihyperglycemic and antihyperlipidemic activities of aqueous extract of *Hericium erinaceus* in experimental diabetic rats. BMC Complement Altern Med. (2013) 13:253. 10.1186/1472-6882-13-253 24090482 PMC3852124

[9] Mendel F Chemistry, nutrition, and health-promoting properties of *Hericium erinaceus* (lion's mane) mushroom fruiting bodies and mycelia and their bioactive compounds. J Agric Food Chem. (2015) 63:7108–23. 10.1021/acs.jafc.5b02914 26244378

[10] Ahmad A Anjum FM Zahoor T Nawaz H Din A Physicochemical and functional properties of barley β-glucan as affected by different extraction procedures. Int J Food Sic Technol. (2010) 44:181–7. 10.1111/j.1365-2621.2008.01721.x

[11] Izydorczyk MS Biliaderis CG Macri LJ Macgregor AW Fractionation of Oat (1→3), (1→4)-β-D-glucans and characterisation of the fractions. J Cereal Sic. (1998) 27:321–5. 10.1006/jcrs.1997.0166

[12] Danielson ME Dauth R Elmasry NA Langeslay RR Magee AS Will PM. Enzymatic method to measure β-1,3-β-1,6-glucan content in extracts and formulated products (GEM assay). J Agric Food Chem. (2010) 58:10305. 10.1021/jf102003m 20809622

[13] Nyman AAT Aachmann FL Rise F Ballance S Samuelsen ABC. Structural characterization of a branched (1→6)-α-mannan and β-glucans isolated from the fruiting bodies of *Cantharellus cibarius*. Carbohydr Polym. (2016) 146:197–207. 10.1016/j.carbpol.2016.03.052 27112866

[14] Li WL Zheng HC Bukuru J Kimpe ND. Natural medicines used in the traditional Chinese medical system for therapy of diabetes mellitus. J Ethnopharmacol. (2004) 92:1–21. 10.1016/j.jep.2003.12.031 15099842

[15] Tosh S Bordenave N. Emerging science on benefits of whole grain oat and barley and their soluble dietary fibers for heart health, glycemic response, and gut microbiota. Nutr Rev. (2020) 78:13–20. 10.1093/nutrit/nuz085 32728756

[16] Englyst H Kingman S Cummings J. Classification and measurement of nutritionally important starch fractions. Eur J Clin Nutr. (1992) 46 (Suppl. 2):S33–50. 1330528

[17] Truswell A Thomas B Brown A. Survey of dietary policy and management in British diabetic clinics. Br Med J. (1975) 4:7–11. 10.1136/bmj.4.5987.7 1174929 PMC1674774

[18] Chen B Han J Li B Wenzhao W Ke M Hongwei L. Identification and α-glucosidase inhibitory activity of meroterpenoids from *Hericium erinaceus*. Planta Med. (2020) 86:571–8. 10.1055/a-1146-8369 32325508

[19] Han Z Ye J Wang G. Evaluation of *in vivo* antioxidant activity of *Hericium erinaceus* polysaccharides. Int J Biol Macromol. (2013) 52:66–71. 10.1016/j.ijbiomac.2012.09.009 23000690

[20] Sasaki T Kohyama K. Influence of non-starch polysaccharides on the *in vitro* digestibility and viscosity of starch suspensions. Food Chem. (2012) 133:1420–6. 10.1016/j.foodchem.2012.02.029 23140698

[21] Kim H White P. Impact of the molecular weight, viscosity, and solubility of β-glucan on *in vitro* oat starch digestibility. J Agric Food Chem. (2013) 61:3270–7. 10.1021/jf305348j 23469761

[22] Granfeldt Y Bjrck I Drews A Tovar J. An *in vitro* procedure based on chewing to predict metabolic response to starch in cereal and legume products. Eur J Clin Nutr. (1992) 46:649–60. 1396482

[23] Castro-Alves V Nascimento J. α- and β-D-Glucans from the edible mushroom *Pleurotus albidus* differentially regulate lipid-induced inflammation and foam cell formation in human macrophage-like THP-1 cells. Int J Biol Macromol. (2018) 111:1222–8. 10.1016/j.ijbiomac.2018.01.131 29366884

[24] Zhang A Deng Y Sun P Meng X Zhang J Structural elucidation of a neutral water-soluble α-D-glucan from the fungus of *Hericium erinaceus*. J Food Biochem. (2011) 35:1680–5. 10.1111/j.1745-4514.2010.00492.x

[25] Semedo M Karmali A Fonseca L. A high throughput colorimetric assay of β-1,3-D-glucans by Congo red dye. J Microbiol Methods. (2015) 109:140–8. 10.1016/j.mimet.2014.12.020 25555819

[26] Lin J Lin Z Yi SU Huang J Chen S Lin H Purification and structure analysis of β-glucan from *Pleurotus eryngii* cell wall. Chin J Trop Crops. (2013) 34:1825–30. 10.3969/j.issn.1000-2561.2013.09.035

[27] Zhang X Kong X Hao Y Zhang X Zhu Z. Chemical structure and inhibition on α-glucosidase of polysaccharide with alkaline-extracted from glycyrrhiza inflata residue. Int J Biol Macromol. (2020) 147:1125–35. 10.1016/j.ijbiomac.2019.10.081 31739069

[28] Kocharova N Hatano K Shaskov A Knirel Y Kochetkov N Pier G. The structure and serologic distribution of an extracellular neutral polysaccharide from *Pseudomonas aeruginosa* immunotype 3. J Biol Chem. (1989) 264:15569–73. 2504722

[29] Zhuang H Chen Z Feng T Yang Y Zhang J Liu G. Characterization of Lentinus edodes β-glucan influencing the *in vitro* starch digestibility of wheat starch gel. Food Chem. (2017) 224:294–301. 10.1016/j.foodchem.2016.12.087 28159269

[30] Panda B Maity P Nandi A Pattanayak M Manna D Mondal S. Heteroglycan of an edible mushroom *Pleurotus cystidiosus*: structural characterization and study of biological activities. Int J Biol Macromol. (2017) 95:833–42. 10.1016/j.ijbiomac.2016.11.121 27932258

[31] Feng T Shui M Chen Z Zhuang H Wang W Yang Y *Hericium Erinaceus* β-glucan modulates *in vitro* wheat starch digestibility. Food Hydrocoll. (2019) 96:424–32. 10.1016/j.foodhyd.2019.05.044

[32] Iwata F Mizuguchi Y Ko H Ushiki T. Nanomanipulation of biological samples using a compact atomic force microscope under scanning electron microscope observation. J Electron Microsc. (2011) 60:359–66. 10.1093/jmicro/dfr070 22049270

[33] Xiao Z Zhou W Zhang Y. Fungal polysaccharides. Adv Pharmacol. (2020) 87:277–99. 10.1016/bs.apha.2019.08.003 32089236

[34] Gieroba B Sroka-Bartnicka A Kazimierczak P Kalisz G Lewalska-Graczyk A Vivcharenko V. Spectroscopic studies on the temperature-dependent molecular arrangements in hybrid chitosan/1,3-β-D-glucan polymeric matrices. Int J Biol Macromol. (2020) 159:911–21. 10.1016/j.ijbiomac.2020.05.155 32445816

[35] Ookushi Y Sakamoto M Azuma J Extraction of β-glucan from the water-insoluble residue of hericium erinaceum with combined treatments of enzyme and microwave irradiation. J Appl Glycosci. (2008) 55:225–9. 10.5458/jag.55.225

[36] Agbenorhevi JK Kontogiorgos V Kirby AR Morris VJ Tosh SM. Rheological and microstructural investigation of oat β-glucan isolates varying in molecular weight. Int J Biol Macromol. (2011) 49:369–77. 10.1016/j.ijbiomac.2011.05.014 21640753

[37] Akine S Miyashita M Piao S Nabeshima T Perfect encapsulation of a guanidinium ion in a helical trinickel(II) metallocryptand for efficient regulation of the helix inversion rate. Inorg Chem Front. (2014) 1:53–7. 10.1039/C3QI00067B

[38] Zhang Y Zhang H Wang L Qian H Qi X Ding X. The effect of oat β-glucan on *in vitro* glucose diffusion and glucose transport in rat small intestine. J Sic Food Agric. (2015) 96:484–91. 10.1002/jsfa.7114 25639602

[39] Regand A Chowdhury Z Tosh SM Wolever TMS Wood P. The molecular weight, solubility and viscosity of oat beta-glucan affect human glycemic response by modifying starch digestibility. Food Chem. (2011) 129:297–304. 10.1016/j.foodchem.2011.04.053 30634230

[40] Liu Y Zhao Y Yang Y Tang Q Zhou S Wu D Structural characteristics and hypoglycemic activity of polysaccharides from Coprinus comatus. Bioact Carbohydr Diet Fibre. (2013) 2:164–9. 10.1016/j.bcdf.2013.10.001

